# Waterborne Phosphated Alkynediol-Modified Mica Nanosheet/Acrylic Nanocomposite Coatings with Superior Anticorrosive Performance

**DOI:** 10.3390/nano15161266

**Published:** 2025-08-16

**Authors:** Rui Yuan, Zhixing Tang, Mindi Xiao, Minzhao Cai, Xin Yuan, Lin Gu

**Affiliations:** 1 School of Chemical Engineering and Technology, Sun Yat-sen University, Zhuhai 519082, China; yuanrui@mail.sysu.edu.cn (R.Y.); tangzhx23@mail2.sysu.edu.cn (Z.T.); xiaomd3@mail2.sysu.edu.cn (M.X.); 2 Guangdong Pearl River Chemical Industry Coatings Co., Ltd., Zhuhai 519050, China; caiminzhao@gdzjhg.com

**Keywords:** waterborne coating, mica, phosphated alkynediol, corrosion protection enhancement

## Abstract

Mica is a naturally layered material recognized for its superior insulation and exceptional barrier properties; however, it is prone to agglomeration, and its compatibility with resin remains to be resolved. In this work, phosphate butynediol ethoxylate (PBEO), synthesized by the reaction of a commercial corrosion inhibitor, butynediol ethoxylate, with phosphorus pentoxide, was employed to modify mica nanosheets (MNs), as evidenced by FTIR, Raman, and XPS. The obtained MN@PBEO demonstrated improved water dispersibility and enhanced compatibility with acrylic latex. EIS measurements revealed that the impedance (|Z|_0.01Hz_) for the waterborne acrylic coating with 0.5 wt% MN@PBEO was approximately an order of magnitude greater than that of the pure waterborne acrylic coating after 28 days of immersion in a 3.5 wt% NaCl solution. Additionally, compared to the pure waterborne acrylic coating, the 0.5 wt% MN@PBEO/acrylic nanocomposite coating on Q235 carbon steel exhibited a water diffusion coefficient that was roughly ten times lower, demonstrating substantially enhanced corrosion protection, attributable to its superior barrier properties.

## 1. Introduction

Corrosion poses a globally recognized challenge, inflicting detrimental impacts on national economies and industrial sectors [1,2]. To mitigate this issue, diverse effective methods have been developed, including corrosion inhibitors, cathodic protection, electrochemical protection [3], coating protection [4], and the use of alloys [5]. Among these, organic coating technology stands out due to its cost-effectiveness and superior protective performance [6,7,8,9,10]. Waterborne coatings, compared to their traditional solvent-based counterparts, significantly reduce volatile organic compound (VOC) emissions and offer enhanced environmental compatibility [11]. Despite these advantages, their limitations in water and corrosion resistance remain critical concerns [12,13,14,15]. Consequently, augmenting the corrosion resistance of waterborne coatings constitutes a critical challenge. Substantial research indicates that incorporating functional fillers can reinforce the barrier properties of such coatings, thereby elevating their anticorrosion efficacy.

Employing two-dimensional (2D) materials with a “labyrinth effect” as nanofillers offers an optimal approach to enhance coatings’ barrier properties and corrosion resistance [4,16,17,18,19]. Graphene, a typical 2D material known for its exceptional barrier capabilities and chemical stability, has frequently been employed as an ideal protective material for metals in recent years [20,21]. Nevertheless, its high electrical conductivity risks inducing galvanic corrosion, potentially accelerating substrate degradation [22,23]. Consequently, identifying alternative 2D nanofillers with enhanced insulation and barrier performance is crucial in advancing coatings’ corrosion resistance. Mica, a naturally occurring flake silicate, exhibits exceptional physical stability and insulating characteristics. Its layered structure facilitates parallel alignment within coatings, establishing mica nanosheets (MNs) as a viable graphene alternative for corrosion protection [24,25,26]. However, the interface incompatibility between inorganic MNs and organic resins remains a significant challenge. Direct incorporation often causes poor dispersion and agglomeration, generating microcracks and pores that ultimately compromise coating performance.

Therefore, the surface modification of MNs is essential to enhance their dispersion and compatibility within waterborne resins. Cai et al. [27] achieved this by directly grafting epoxy resin onto MNs via high-temperature mechanical ball milling, significantly improving the dispersibility and resin compatibility. However, this method imposes stringent equipment requirements. Wu et al. [28] utilized polyether imide to modify MNs, enabling the electric field-induced oriented distribution in epoxy coatings; the resulting 4 wt% MN/epoxy coating exhibited exceptional corrosion resistance, with a corrosion current density that was six orders of magnitude lower than that of the substrate. Similarly, Ye et al. [4] functionalized MNs with natural tannic acid (TA) through catechol–OH interactions (MN@TA), where 0.5 wt% loading notably enhanced the barrier properties and corrosion resistance of waterborne epoxy coatings. Nevertheless, research on MN modification for waterborne acrylic latex remains scarce. Mica’s ready availability, layered silicate architecture, and smaller environmental footprint relative to synthetic substitutes such as boron nitride nanosheets (BNNSs) deliver clear sustainability benefits. Moreover, its intrinsic hydrophilicity and hydroxyl-dense surface enable straightforward functionalization, thereby reducing the need for energy-intensive processing stages. In this work, we utilize butynediol ethoxylate (BEO)—an inexpensive, commercial corrosion inhibitor—to functionalize MNs through phosphate groups, markedly boosting the anticorrosion performance of waterborne acrylic coatings.

**Figure 1:**
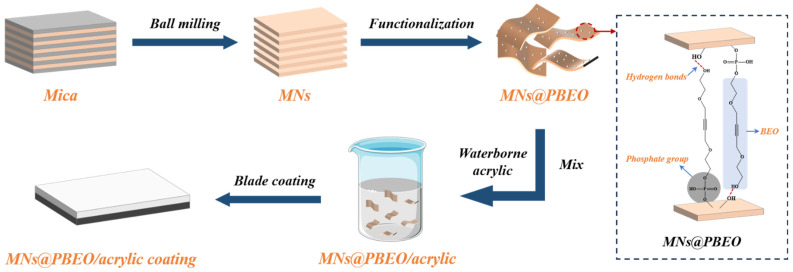
Preparation schematic for MN@PBEO and waterborne acrylic nanocomposite coating.

BEO—an alkyldiol bearing two reactive hydroxyls—forms hydrogen bonds with the hydroxyl and oxygen sites on MNs, enlarging the intersheet spacing while curbing aggregation. The PBEO adduct, generated by reacting BEO with phosphorus pentoxide, supplies phosphate groups that chelate metal surface hydroxyls, yielding a denser passivation layer that markedly retards corrosion [29]. Consequently, MN@PBEO exhibits excellent water dispersibility and compatibility with acrylic latex (Figure 1). After 28 days in 3.5 wt% NaCl, EIS shows |Z|_0.01Hz_ for 0.5 wt% MN@PBEO coatings—roughly ten-fold that of the pristine waterborne acrylic. On Q235 steel, the composite’s water diffusion coefficient is also an order of magnitude lower, evidencing superior barrier performance rooted in mica’s aligned, tortuous mineral layers.

## 2. Results and Discussion

### 2.1. Preparation and Characterization of MN@PBEO

**Figure 2:**
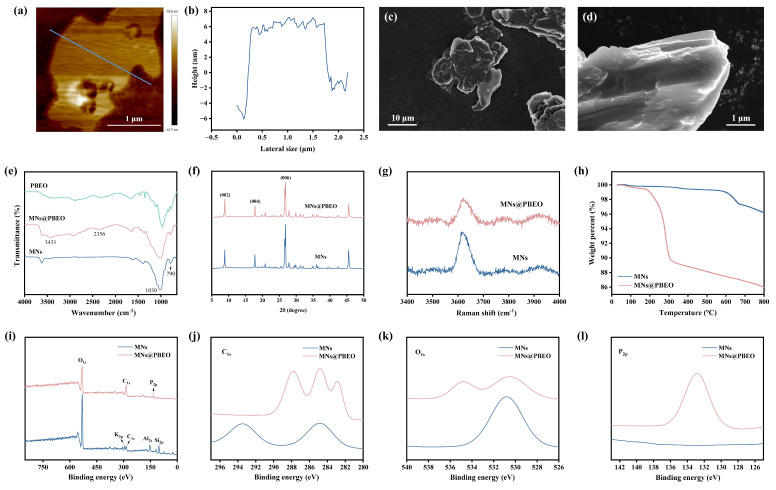
(**a**,**b**) AFM image and surface height map of MN@PBEO. (**c**,**d**) SEM images of MN@PBEO. (**e**) FT-IR, (**f**) XRD, (**g**) Raman, and (**h**) TGA of MNs and MN@PBEO. (**i**) XPS full spectra, (**j**) C_1s_, (**k**) O_1s_, and (**l**) P_2p_ plots of MNs and MN@PBEO.

The synthesis process of MN@PBEO is illustrated in Figure 1, with a detailed experimental section provided in the Supporting Information. Atomic force microscopy (AFM) and scanning electron microscopy (SEM) characterized the surface morphology, as shown in Figure 2. AFM images of the MN@PBEO nanosheets are shown in Figure 2a,b, accompanied by the corresponding height profiles. The analysis reveals that their thickness is around 8–9 nm. Figure 2c,d display the SEM images of MN@PBEO, indicating that the MN@PBEO retains an irregular flake morphology, which suggests that the layered structure of the MNs was preserved during the modification process. Additionally, the MN@PBEO nanosheets exhibit the same dimensions as the original MNs.

The modified MN@PBEO was analyzed using Fourier transform infrared spectroscopy (FTIR), as shown in Figure 2e. Both MNs and MN@PBEO exhibit characteristic absorption peaks at 1030 cm^−1^ and 790 cm^−1^, which correspond to the stretching and bending vibrations of Si–O, respectively. Additionally, several new and distinct absorption peaks are observed in MN@PBEO compared to MNs. Notably, an absorption peak corresponding to the stretching vibration of hydroxyl groups is identified at 3431 cm^−1^, while a stretching vibration absorption peak for methylene C–H is detected in the range of 2879–2956 cm^−1^, and a stretching vibration absorption peak for C≡C is noted at 2356 cm^−1^. Although the absorption peaks associated with P–O–H (900–1050 cm^−1^), P=O stretching vibrations (1130 cm^−1^), and the stretching vibration characteristic of P–O–C (1090 cm^−1^) overlap with the Si–O absorption peak, the presence of hydroxyl and C≡C absorption peaks provide evidence of the successful modification of MNs using PBEO.

As shown in Figure 2f, X-ray diffraction (XRD) was employed to study the crystal structure of MN@PBEO. Both MNs and MN@PBEO exhibited diffraction peaks corresponding to the (002), (004), and (006) planes, indicating that PBEO did not alter the crystalline structure of the MNs. However, a noticeable reduction in the intensity of the (006) peak was observed for MN@PBEO compared to the unmodified MNs, suggesting a decrease in crystallinity following modification. Additionally, MN@PBEO exhibited larger (006) crystal plane spacing than the MNs, indicating the further exfoliation of the MNs due to PBEO modification. Figure 2g displays the Raman spectra of the MNs and MN@PBEO, with *E*_2g_ peaks displayed at 3620 cm^−1^. Following modification, the marked attenuation of the *E*_2g_ peak signals reflects weakened intersheet interactions and expanded lamellar spacing imparted by PBEO [30].

Furthermore, thermal gravimetric analysis (TGA) and X-ray photoelectron spectroscopy (XPS) were employed to characterize the PBEO-modified MNs. In Figure 2h, the TGA results reveal that the MNs exhibited minimal mass loss during the heating process, with an overall loss of 3.78%. The weight loss observed below 100 °C (approximately 0.12%) is primarily attributed to the desorption of moisture adsorbed on the surfaces of the MNs. Following 100 °C, the mass of the MNs remained relatively stable until the temperature surpassed 600 °C, at which point a secondary mass loss commenced. By comparison, MN@PBEO commenced decomposition at 160 °C and, upon heating to 800 °C, exhibited a 13.99% mass loss—substantially higher than that of the pristine MNs. This additional loss is chiefly attributable to PBEO pyrolysis.

The XPS spectra for the MNs and MN@PBEO are displayed in Figure 2i–l, including C_1s_, O_1s_, and P_2p_ spectra. The comprehensive XPS spectra show a significant increase in the carbon and phosphorus content in the MN@PBEO sample compared to the MNs. Specifically, the relative content of C_1s_ rose from 11.08% to 45.21%, while the relative content of P_2p_ increased from 0.24% to 7.29% (Table S1). All the above results further substantiate the successful modification of MNs by PBEO.

**Figure 3:**
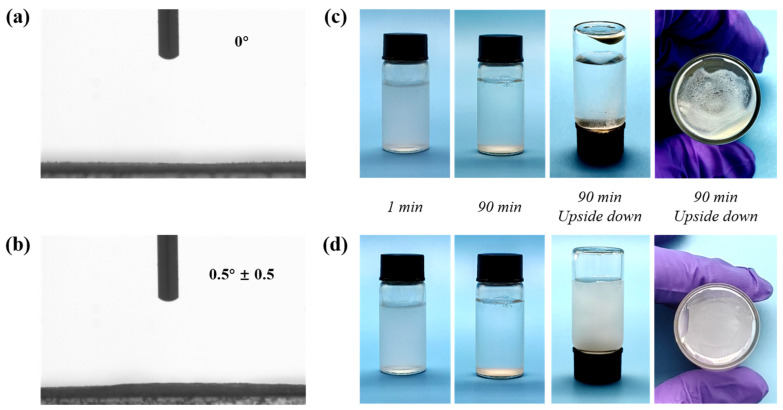
(**a**,**b**) Water contact angles of MNs and MN@PBEO. (**c**,**d**) Dispersion images of MNs and MN@PBEO.

Water contact angle measurements on MNs and MN@PBEO were performed to confirm PBEO’s influence on the wetting behavior of MNs. In Figure 3a,b, the MNs display a pronounced hydrophilic nature, with a water contact angle approaching 0°. Furthermore, the incorporation of PBEO, which is a hydrophilic substance, did not influence the surface water contact angle of the MNs.

As illustrated in Figure 3c,d, MNs and MN@PBEO, both at a concentration of approximately 0.8 mg/mL, were prepared in identical conditions before being left to stand for 90 min. The unmodified MNs showed sedimentation after 90 min, with the inversion of the container revealing the agglomeration of the nanosheets at the bottom. In contrast, although the modified MN@PBEO samples also exhibited some sedimentation after 90 min, they could be redispersed in water upon inverting the container. This indicates the effective functionalization of MNs using PBEO, and their dispersion was notably improved.

### 2.2. Morphologies of the MN@PBEO/Acrylic Coatings

**Figure 4:**
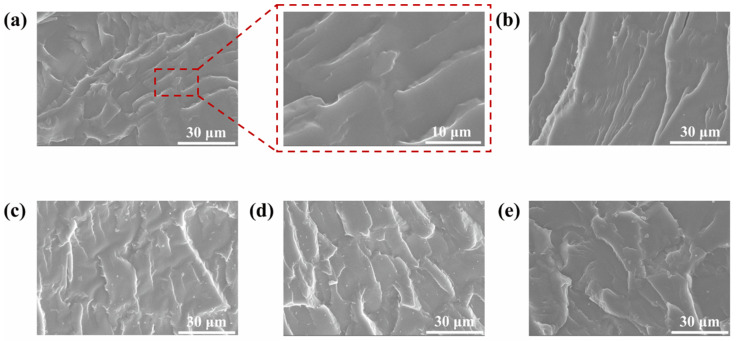
SEM images of MN@PBEO/acrylic composite coatings section at various loadings of (**a**) 0 wt%; (**b**) 0.2 wt%; (**c**) 0.5 wt%; (**d**) 1 wt%; (**e**) 2 wt%.

Figure 4 presents images of composite coatings with different mass fractions, as observed through SEM. The pure waterborne acrylic coating shows cracks running parallel to the propagation direction. At 0.2 wt% MN@PBEO, this pattern persists, yet higher loadings progressively replace the cracks with wrinkled fracture surfaces. The emergence of these wrinkles suggests that the MN@PBEO nanofillers contribute significantly to the toughening of the composite resin. These wrinkles facilitate additional deformation pathways under stress, thereby enhancing the material’s ability to absorb fracture energy and diminishing the probability of fracture occurrence. In addition, as shown in Figure S1, the adhesion properties of the pure waterborne acrylic coating and composite coating were evaluated by the pull-off method. The appropriate incorporation of MN@PBEO led to an enhancement in the adhesion of the composite coating, achieving a value of 4.0 MPa, in contrast to 3.5 MPa for the pure waterborne acrylic coating. In addition, the water contact angles for the MN@PBEO/MMA-BA composite coatings were measured at various loadings (Figure S2). The pristine MMA-BA coating had an angle of ~81.3°, revealing its hydrophilic nature. Incorporating MN@PBEO progressively lowered the contact angle, reflecting the additive’s hydrophilic contribution. At 1 wt% MN@PBEO, however, the angle rebounded to 81.0°, presumably because the polymer’s reduced free volume counteracted the hydrophilic effect.

### 2.3. Corrosion Protection Performance of MN@PBEO/Acrylic Composite Coatings

#### 2.3.1. Electrochemical Characterization

**Figure 5:**
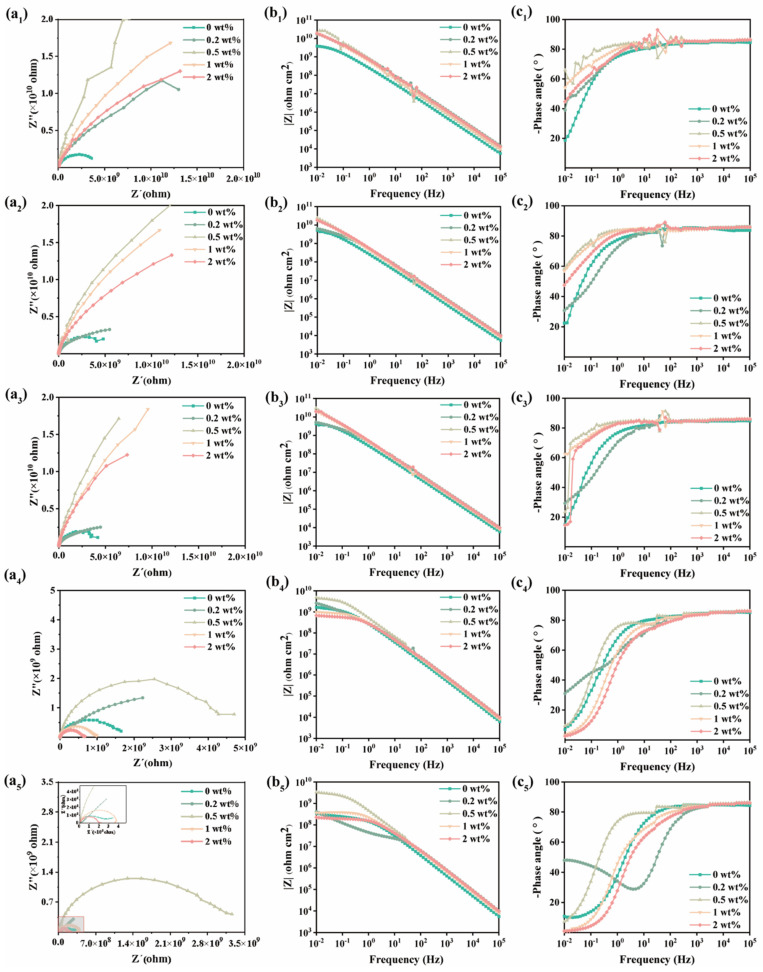
EIS plots of MN@PBEO/acrylic composite coatings on Q235 carbon steel ((**a**) is Nyquist plot, (**b**,**c**) are Bode plots, and the subscript numbers 1/2/3/4/5 represent the durations of immersion in 3.5 wt% NaCl aqueous solution: 1 d/7 d/14 d/21 d/28 d, respectively).

Electrochemical impedance spectroscopy (EIS) was employed to evaluate the MN@PBEO/acrylic composite coatings at different loadings; the resulting Nyquist and Bode plots were analyzed. The impedance modulus at 0.01 Hz (|Z|_0.01Hz_) is a key EIS parameter that reflects corrosion resistance, with larger capacitive arc diameters generally corresponding to superior anticorrosion performance [31]. As presented in Figure 5, after 1 day of immersion, the pure waterborne acrylic coating demonstrated a significant capacitive arc radius in the Nyquist plot, with a |Z| of 4.71 × 10^9^ Ω·cm^2^ at a frequency of 0.01 Hz. This finding suggests that, during the initial immersion period, the waterborne acrylic coating effectively inhibited the majority of corrosive agents from making direct contact with the underlying metal substrate, thereby providing a protective barrier. However, as the immersion duration increased to 28 days, the progressive infiltration of the corrosive substances resulted in a reduction in the |Z|_0.01Hz_ value for the waterborne acrylic coating to 3.49 × 10^8^ Ω·cm^2^, indicating the deterioration of the coating’s protective efficacy. For the MN@PBEO/acrylic coatings at different loadings, after 1 day of immersion, the |Z|_0.01Hz_ values recorded for the 0.2 wt%, 0.5 wt%, 1 wt%, and 2 wt% MN@PBEO/acrylic composite coatings were 1.67 × 10^10^ Ω·cm^2^, 2.64 × 10^10^ Ω·cm^2^, 2.07 × 10^10^ Ω·cm^2^, and 1.85 × 10^10^ Ω·cm^2^, respectively. The Bode plot analysis demonstrates that the curves for all composite coatings exhibit a linear relationship, with a slope of approximately −1. This observation indicates that, at this stage, all composite coatings possessed commendable barrier properties, effectively limiting the ingress of corrosive agents and the progression of corrosion mechanisms beneath the coating.

Following 28 days of extended immersion, the |Z|_0.01Hz_ values for all composite coatings exhibited a noticeable decline. Specifically, the values for the 0.2 wt%, 1 wt%, and 2 wt% MN@PBEO/acrylic nanocomposite coatings decreased to 4.12 × 10^8^ Ω·cm^2^, 3.64 × 10^8^ Ω·cm^2^, and 2.24 × 10^8^ Ω·cm^2^, respectively. The 0.2 wt% MN@PBEO/acrylic composite coating displayed a diffusion tail in the Nyquist plot, indicating that corrosive media had penetrated the coating and reached the interface between the coating and the metal substrate. This finding implies that the concentration of MN@PBEO nanosheets incorporated was insufficient to significantly enhance the corrosion resistance of the waterborne acrylic coating. Conversely, an excessive amount of MN@PBEO nanosheets (1 wt% and 2 wt%) can further reduce the free volume of the polymer, affecting the crosslinking during the coating drying process, and cannot enhance the corrosion resistance of the composite coating. In contrast, the 0.5 wt% MN@PBEO/acrylic composite coating exhibits the best corrosion resistance, with its |Z|_0.01Hz_ value only decreasing to 3.29 × 10^9^ Ω·cm^2^ after 28 days of immersion, showing a small change in the impedance value. This indicates that an appropriate amount of MN@PBEO (0.5 wt%) can slow down the penetration rate of the corrosive medium, effectively improving the corrosion resistance of the coating. In summary, by adjusting the mass fraction of MN@PBEO, the corrosion resistance of waterborne acrylic coatings can be effectively improved, with the 0.5 wt% MN@PBEO/acrylic composite coating showing the best corrosion resistance on Q235 carbon steel, while the corrosion resistance is not enhanced with either a small or excessive amount of MN@PBEO.

**Figure 6:**
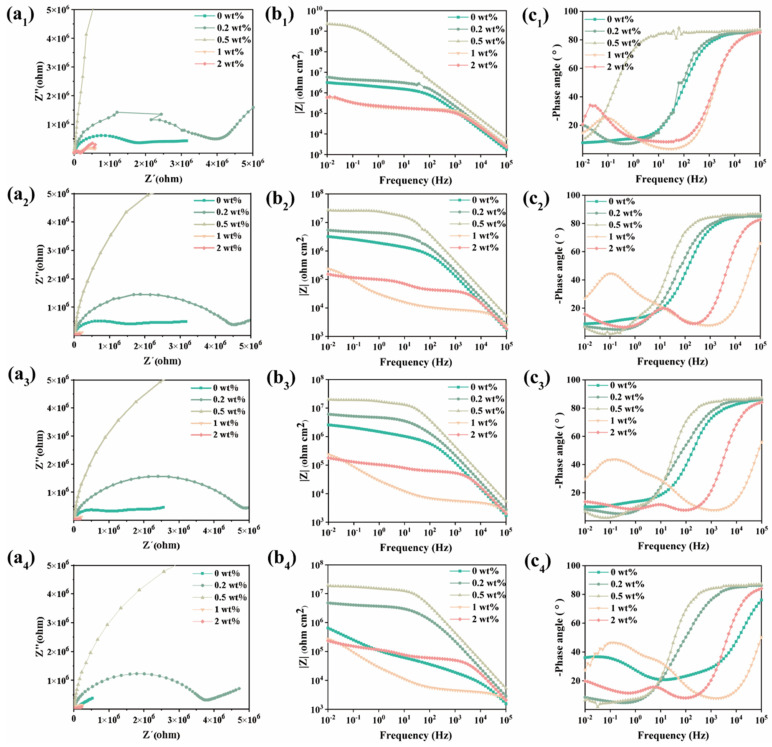
Nyquist (**a**) and Bode (**b**,**c**) plots of MN@PBEO/acrylic coatings on aluminum alloy. (The subscript numbers 1/2/3/4 represent the durations of immersion in 3.5 wt% NaCl aqueous solution: 1 d/7 d/14 d/21 d, respectively).

Figure 6 illustrates the Nyquist and Bode plots of the composite coatings applied to 5052 aluminum alloy with varying mass fractions. After 1 day of immersion, the |Z|_0.01Hz_ value of the pure waterborne acrylic coating was 4.41 × 10^6^ Ω·cm^2^, exhibiting a plateau in the low-frequency region and extending to the mid-frequency region. This indicates that the electrochemical reaction under the coating had already commenced by this time. The composite coating containing 0.5 wt% MN@PBEO exhibits markedly enhanced corrosion protection performance, particularly in terms of the arc diameter, which is considerably larger than that observed in the pure waterborne coating, and the |Z|_0.01Hz_ value is two orders of magnitude higher than that of the pure waterborne coating, indicating that the appropriate addition of MN@PBEO effectively improves the protection capabilities of composite coatings for 5052 aluminum alloy. However, for the waterborne acrylic composite coatings with 1 wt% and 2 wt% MN@PBEO, the |Z|_0.01Hz_ value drops below 10^6^ Ω·cm^2^ on the first day of immersion, indicating that these composite coatings have lost their protective effects on the base metal at the initial stage. This is due to the fact that the addition of excess MN@PBEO nanosheets affects the crosslinking of the coating during the drying process, thus reducing the overall corrosion protection of the coating. Moreover, the pure waterborne acrylic coating had completely lost its protective effect on the base metal with increasing immersion times at up to 21 days. However, the |Z|_0.01Hz_ values of the waterborne acrylic composite coatings with 0.2 wt% and 0.5 wt% MN@PBEO were 4.77 × 10^6^ Ω·cm^2^ and 1.95 × 10^7^ Ω·cm^2^, respectively, even after a longer immersion time, indicating that the composite coatings were still able to provide some protection to the substrate metal, even though corrosive media had already started to reach the metal/coating interface. Overall, the addition of MN@PBEO improved the corrosion resistance of the waterborne acrylic composite coatings on 5052 aluminum alloy, with the best corrosion resistance achieved at 0.5 wt% MN@PBEO.

The phosphate groups in ANPEO_10_-P_1_ undergo chelation with the Fe-OH groups on the surface of Q235 steel, initiating the formation of an iron phosphate passivation layer [29]. Meanwhile, the crosslinking reaction between phosphate groups gradually enhances the densification of the coating. In particular, the synergistic effect of physical barriers and chemical passivation explains the excellent long-term corrosion resistance of the surface of Q235 carbon steel, and its iron phosphate interface shows better stability compared with the 5052 aluminum matrix. Meanwhile, alumina (Al^3+^) and phosphate groups in the copolymer (–PO_4_^3−^) can also form Al–O–P covalent bonds; however, the bonding strength and stability of these bonds are inferior to those found in the iron oxide (Fe^3+^) system. Therefore, the coating’s anticorrosion mechanism on 5052 aluminum alloy is primarily attributed to the physical barrier effect of the coating.

**Figure 7:**
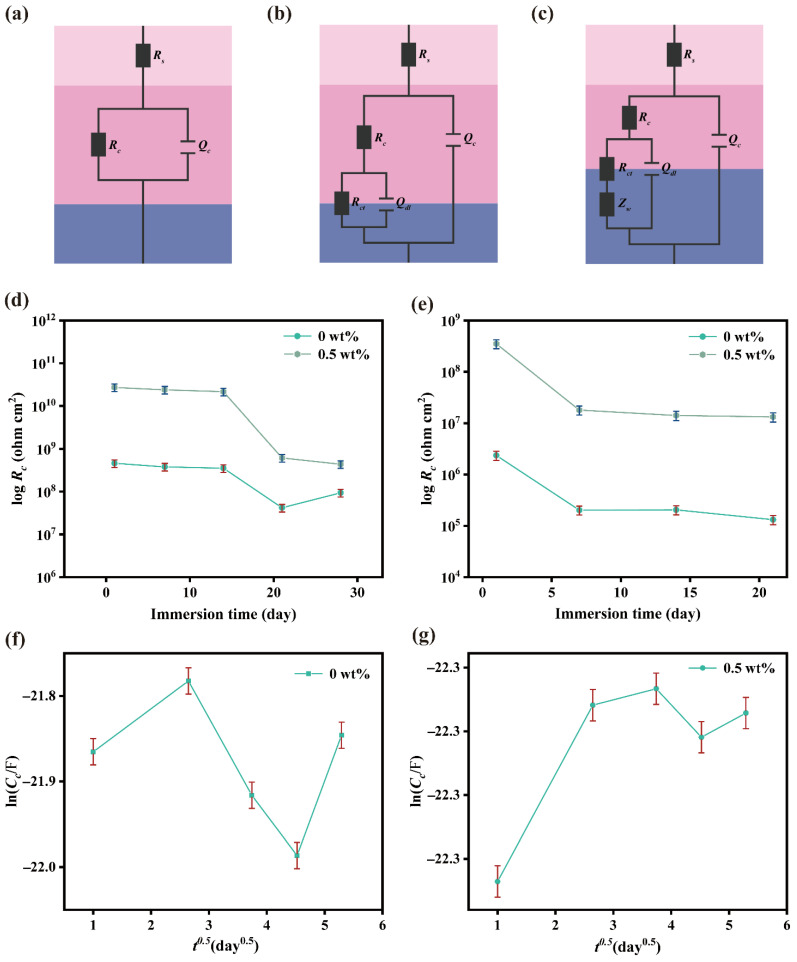
(**a**–**c**) The equivalent circuits of MN@PBEO/acrylic composite coatings. (**d**) The *R_c_* changes of pure acrylic and 0.5 wt% MN@PBEO/acrylic composite coatings on Q235 steel and (**e**) aluminum alloy in 3.5 wt% NaCl solution. (**f**) The capacitance *C* changes of the pure acrylic coating and (**g**) 0.5 wt% MN@PBEO/acrylic coatings on Q235 steel in 3.5 wt% NaCl solution [error bars: ± SD, n = 3].

In order to analyze the corrosion mechanism of the coating at different stages, simulations were conducted using the ZSimpWin software. Three distinct equivalent circuit models were employed to illustrate the alterations in the corrosion protection characteristics of the coatings at varying stages (Figure 7a–c). In the figure, *R_c_* denotes the coating resistance [32], *R_ct_* signifies the charge transfer resistance [33], *R_s_* represents the solution resistance, *Z_w_* denotes the Warburg impedance [34], *Q_c_* is the constant phase element of coating capacitance, and *Q_dl_* represents the double-layer capacitance between the coating and metal interface. From model A to model C, in the initial phase of immersion, the coating served as an insulating barrier, effectively preventing the penetration of corrosive substances and protecting the metal substrate from corrosion. Moreover, with the increase in the immersion time, a diffusion process occurred near the electrode; subsequently, the corrosion reaction rate of the base metal became accelerated.

Figure 7d,e show that, on both Q235 carbon steel and 5052 aluminum alloy, the 0.5 wt% MN@PBEO/acrylic coating exhibits a higher *R*_c_ value than the pure waterborne acrylic coating. Because *R*_c_ directly reflects barrier performance, the composite consistently outperforms the neat coating. After 28 days of immersion, the composite’s *R*_c_ remains at 1.0 × 10^10^ Ω·cm^2^—almost two orders of magnitude above that of the acrylic control (1.0 × 10^8^ Ω·cm^2^)—confirming that the judicious addition of MN@PBEO nanosheets markedly improves the barrier properties. The same two-orders-of-magnitude superiority is observed on 5052 aluminum alloy.

Moreover, capacitance–time measurements were performed to probe water ingress through the coating, using the water diffusion coefficient as the barrier performance indicator. The analysis focused on the 10 kHz capacitance, calculated from the following equation, where *f* denotes the frequency, |*Z*| represents the impedance modulus, and *θ* is the phase angle [35]:

(1) $$C=\frac{1}{2\pi f|Z|sin\theta }$$

The ln*C_c_*-*t*^0.5^ curves for the pure waterborne coating and the 0.5 wt% MN@PBEO/acrylic composite coating, after immersion in a 3.5 wt% sodium chloride aqueous solution, are illustrated in Figure 7f,g. Here, *C_c_* represents the capacitance of the undamaged coating. The neat acrylic coating shows an initial rise in ln*C*_c_ followed by a decline after about eight days, likely caused by the leaching of organic constituents. In contrast, the 0.5 wt% MN@PBEO/acrylic coating displays a linear ln*C*_c_ increase that adheres to Fick’s first law [35], indicating uniform moisture diffusion. Prolonged soaking kept ln*C*_c_ steady until water sorption saturated the film, after which water started penetrating the coating–substrate interface. Assuming negligible coating swelling, the diffusion coefficient D can be calculated as

(2) $$D=\frac{{b_{s}}^{2}L^{2}\pi }{4(lnC_{\infty }-lnC_{0}{)}^{2}}$$

In the equation, *C*_0_ is the initial capacitance measured while immersed, $C_{\infty }$ denotes the coating capacitance at water saturation, L is the coating thickness, and *b_s_* is the slope of ln*C_c_*-*t*^0.5^. Calculations show that, for Q235 carbon steel, the 0.5 wt% MN@PBEO/acrylic composite coating exhibits a water diffusion coefficient of (5.787 ± 0.145) × 10^−14^ m^2^/s (Table 1), an order of magnitude lower than that of the neat waterborne acrylic coating. This result demonstrates that incorporating 0.5 wt% MN@PBEO lengthens the path for corrosive species and lowers the water diffusion coefficient, thereby enhancing the composite coating’s barrier performance.

#### 2.3.2. Morphologies of MN@PBEO/Acrylic Composite Coatings After Immersion

**Figure 8:**
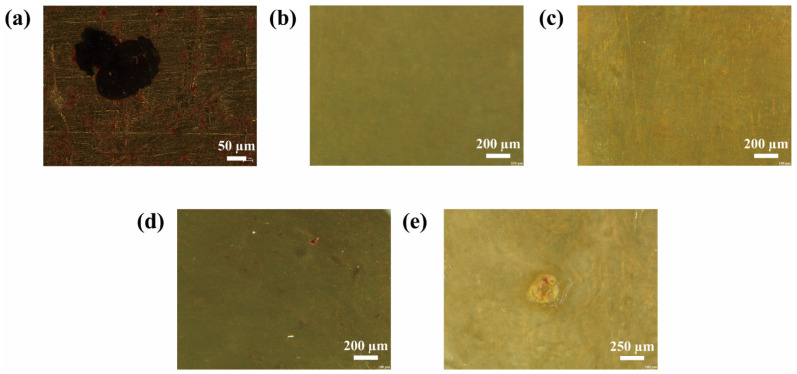
Surface morphologies of various coatings on Q235 steel after immersion for 20 days. ((**a**–**e**) denote 0 wt%, 0.2 wt%, 0.5 wt%, 1 wt%, and 2 wt% MN@PBEO/acrylic coatings, respectively).

**Figure 9:**
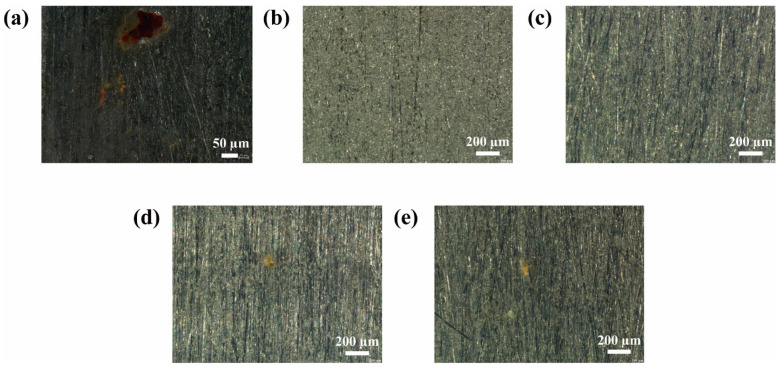
Surface morphologies of various coatings on aluminum alloy after immersion for 20 days. ((**a**–**e**) denote 0 wt%, 0.2 wt%, 0.5 wt%, 1 wt%, and 2 wt% MN@PBEO/acrylic coatings, respectively).

Figure 8 and Figure 9 present the corrosion morphologies of different coatings after 20 days of immersion in 3.5 wt% NaCl solution on Q235 carbon steel and 5052 aluminum alloy, respectively. On Q235 carbon steel, visible corrosion appeared on the 1 wt%, 2 wt%, and neat waterborne acrylic films, indicating that excessive MN@PBEO is detrimental rather than beneficial. Conversely, the 0.2 wt% and 0.5 wt% MN@PBEO/acrylic coatings remained almost intact, confirming that the optimum loading of MN@PBEO markedly improves the corrosion resistance of films on this substrate. Similar trends were observed on 5052 aluminum alloy: corrosion occurred on the 1 wt%, 2 wt%, and neat acrylic coatings, whereas the 0.2 wt% and 0.5 wt% composite coatings showed no attack, owing to the barrier action of the MN@PBEO nanosheets. These visual findings align with the EIS data and further verify that 0.5 wt% MN@PBEO substantially boosts the protective performance of waterborne acrylic coatings on both Q235 carbon steel and 5052 aluminum alloy.

#### 2.3.3. Corrosion Protection Mechanism of MN@PBEO/Acrylic Composite Coatings

**Figure 10:**
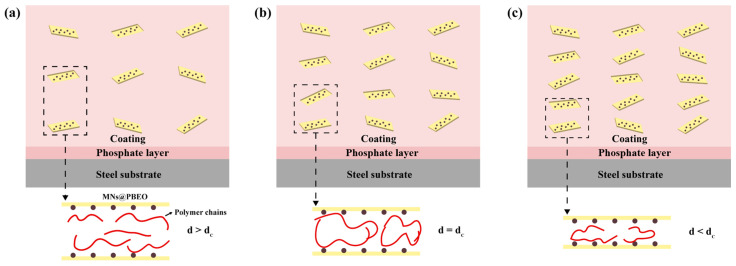
Anticorrosive mechanism of MN@PBEO/acrylic coatings (MN@PBEO addition amount: (**a**) very little; (**b**) Appropriate amount; (**c**) Excessive).

The anticorrosion mechanism was elucidated by systematically analyzing the electrochemical responses of MN@PBEO composite coatings with varying loadings during immersion tests, as depicted in Figure 10. When the neat waterborne acrylic latex—devoid of nanosheets—was applied to Q235 carbon steel and 5052 aluminum alloy, its phosphate groups reacted with the surface hydroxyl groups to generate a passivation layer that isolated the substrates from corrosive species and retarded corrosion. Introducing only a trace amount of MN@PBEO leaves the polymer chains sufficiently mobile, with the intermolecular gaps still being large; hence, the corrosion resistance hardly improves relative to the pristine acrylic coating (Figure 10a). At an optimal concentration, MN@PBEO nanosheets occupy the free volume within the polymer, restricting segmental motion and sharply increasing the coating’s barrier performance. Here, the composite coating and underlying passivation layer act jointly as a physical shield against aggressive media. Moreover, the uniformly dispersed nanosheets densify the film, creating a “labyrinth effect” that forces corrosive species to travel an extended path, further boosting the protection (Figure 10b). However, once the nanosheet fraction surpasses the optimum, the excessive filler constrains the polymer free volume, disrupts chain entanglement during film formation, and ultimately degrades the corrosion resistance (Figure 10c). Thus, only the properly balanced loading of MN@PBEO can effectively suppress corrosion on Q235 carbon steel and 5052 aluminum alloy surfaces.

## 3. Conclusions

To address the limitations of metal corrosion resistance in waterborne acrylic coatings and the compatibility between MNs and waterborne acrylic latex, we modified MNs using PBEO and added them to acrylic latex to enhance the corrosion resistance. Based on the study presented above, the following conclusions can be inferred.

MN@PBEO demonstrated improved water dispersibility and enhanced compatibility with acrylic latex. After 90 min, the modified MN@PBEO could be redispersed in water upon inverting the container.

The uniform dispersion of MN@PBEO within the coating creates a “labyrinth effect”, providing effective physical shielding and significantly extending the pathway for corrosive media to penetrate the coating. EIS data after immersion confirmed that adding an optimal amount of MN@PBEO markedly enhanced the long-term corrosion protection of the composite coatings. After 28 days in 3.5 wt% NaCl, the 0.5 wt% MN@PBEO/acrylic film retained |Z|_0.01Hz_ values of about 10^9^ Ω·cm^2^ on Q235 carbon steel and 10^7^ Ω·cm^2^ on 5052 aluminum alloy—roughly one order of magnitude greater than those of the neat acrylic coating.

Additionally, the water diffusion coefficient in the 0.5 wt% MN@PBEO/acrylic composite coating on Q235 carbon steel was determined to be one order of magnitude lower than that of the neat waterborne acrylic coating. This pronounced decrease reflects enhanced barrier properties, a key factor in delivering superior corrosion resistance.

This work presents novel ideas for the modification of MNs and their application in acrylic latex, with the objective of enhancing the corrosion resistance of coatings. This advancement aims to promote further development in the field of waterborne acrylic latex.

## Figures and Tables

**Table 1:** Capacitance and water diffusion coefficients *D* of pure acrylic and 0.5 wt% MN@PBEO/acrylic composite coatings on Q235 steel [data format: mean ± SD].

Coating	*lnC*_0_ (F)	*lnC*_∞_ (F)	*L* (μm)	*D* (m^2^/s)
Pure acrylic	−21.87 ± 0.05	−21.85 ± 0.03	123 ± 2	(8.727 ± 0.212) ×10^−13^
0.5 wt% MN@PBEO/acrylic	−22.34 ± 0.07	−22.29 ± 0.04	116 ± 3	(5.787 ± 0.145) ×10^−14^

## Data Availability

The original contributions presented in this study are included in the article/supplementary material. Further inquiries can be directed to the corresponding authors.

## Supplementary Materials

The following supporting information can be downloaded at: https://www.mdpi.com/article/10.3390/nano15161266/s1.
