# Supplementary material for: Waterborne Phosphated Alkynediol-Modified Mica Nanosheet/Acrylic Nanocomposite Coatings with Superior Anticorrosive Performance
Source: Nanomaterials (Basel). 2025 Aug 16;15(16):1266. doi: 10.3390/nano15161266 (PMC12388761; doi:10.3390/nano15161266)
Supplement: Supplementary file 1 [file nanomaterials-15-01266-s001.zip › nanomaterials-3754546-supplementary.pdf]

SUPPORTING INFORMATION

Waterborne Phosphated Alkynediol-Modified Mica Nanosheet/Acrylic Nanocomposite Coatings with Superior Anticorrosive Performance

Rui Yuan [1,†], Zhixing Tang [1,†], Mindi Xiao [1], Minzhao Cai [2], Xin Yuan [1,*] and Lin Gu [1,*]

[1] School of Chemical Engineering and Technology, Sun Yat-sen University, Zhuhai 519082, China; yuanrui@mail.sysu.edu.cn (R.Y.); tangzhx23@mail2.sysu.edu.cn (Z.T.); xiaomd3@mail2.sysu.edu.cn (M.X.)

[2] Guangdong Pearl River Chemical Industry Coatings Co., Ltd., Zhuhai 519050, China; caiminzhao@gdzjhg.com

* Correspondence: yuanx29@mail.sysu.edu.cn (X.Y.); gulin5@mail.sysu.edu.cn (L.G.)

† These authors contributed equally to this work.

1. Experimental section

1.1. Materials

Mica was purchased from Chu Zhou Grea Minerals Co., Ltd. Butynediol ethoxylate was purchased from Shanghai McLean Biochemical Technology Co., Ltd. Phosphorus pentoxide and Polyacrylamide was purchased from Aladdin Reagent (Shanghai) Co., Ltd. Sodium dodecyl diphenyl ether disulfonate (45% aqueous solution) was purchased from Shanghai Yuanye Biotechnology Co., Ltd. Potassium persulfate, sodium bicarbonate, methyl methacrylate (MMA), butyl acrylate (BA) were purchased from Aladdin Reagent (Shanghai) Co., Ltd. 1-allyloxy-3-(4-nonylphenol)-2-propanol polyoxyethylene (10) ether monophosphate ($ANPEO_{10}$-P_1) was provided by Guangzhou ShuangJian Trading Co., Ltd.

1.2 Preparation of PBEO

In a flask, 0.5 mol of BEO was mixed with 1 mol of deionized water. Subsequently, 1 mol of phosphorus pentoxide was added in batches over a period of 30 minutes while stirring continuously. The reaction was conducted at 80°C for 4 h, after which an additional 0.5 mol of BEO was introduced to continue the reaction for another 4 h. Following the reaction, the mixture was dissolved in water, and activated carbon was added for decolorization. The resulting mixture was then filtered to obtain the filtrate, which was subjected to rotary evaporation to yield PBEO.

1.3 Preparation of MNs@PBEO

A total of 25 g of mica, 10 g of polyacrylamide aqueous solution (PAM), and 80 g of ball mill beads were combined in a ball mill tank, maintaining a mass ratio of approximately 2.5:1:8 (mica: medium: ball mill beads). The ball mill was then operated for a duration of 20 h. Following the milling process, deionized water was added to facilitate the separation of the ball mill beads, and the resulting solution was centrifuged for 15 minutes at 2000 rpm. This washing procedure was repeated three times to effectively remove PAM. The resulting wet powder was dried, and the product was collected to yield MNs.

Subsequently, 5 g of MNs and 10 g of PBEO were added to 100 g of deionized water. The mixture underwent continuous ultrasonic treatment for 2 h, with a cycle of closing every 3 seconds and opening every 2 seconds. Following this, the mixture was stirred at 25°C for 18 h to achieve a uniform dispersion of nanosheets. The dispersion was then filtered, washed three times with deionized water, and dried at 100°C for 24 h to obtain MNs@PBEO.

1.4 Preparation of waterborne acrylic latex

The specific procedure involves adding 436.7 g of water and 0.63 g of sodium lauryl diphenyl ether disulfonate, 0.313 g of potassium persulfate, and 1.035 g of sodium bicarbonate into a four-neck round bottom flask. Additionally, 31.3 g of methyl methacrylate and 31.3 g of butyl acrylate are mixed and placed in a constant pressure dropping funnel. The setup includes a straight condenser tube, condensed water, and argon gas, with the temperature raised to 80°C. The constant pressure dripping funnel speed is maintained to complete the 4 h dripping process. Following this, the reaction continues for 1 h at 80°C to yield the seed latex.

Seed latex weighing 53.65 g and potassium persulfate weighing 0.163 g were placed into a four-neck round bottom flask. Methyl methacrylate weighing 59.55 g, butyl acrylate weighing 59.55 g, emulsifier ($ANPEO_{10}$-P_1) weighing 2.526 g, and 75 g of water were combined and emulsified with high-speed shear for 10 minutes to form a pre-latex. The pH of the pre-latex was adjusted to 7 using ammonia water and then transferred to a constant pressure separatory funnel. A straight condenser tube was connected, condensed water and argon gas were introduced, and the temperature was raised to 80°C while maintaining a constant pressure dripping funnel speed to complete the dripping process within 4 h. The heating temperature was then adjusted to 70°C and the reaction continued for 1 h. Subsequently, the heating temperature was lowered to 60°C, a 10 wt% tert-butyl hydroperoxide solution and a 10 wt% FF6 solution were prepared, and added dropwise simultaneously. The reaction was allowed to proceed for 1 h to obtain the waterborne acrylic latex.

1.5 Preparation of MNs@PBEO/acrylic Coatings

The surfaces of Q235 carbon steel and 5052 aluminum alloys were polished using 180 mesh and 600 mesh sandpaper, respectively. Subsequently, the polished steel sheets and aluminum alloys were ultrasonically cleaned in acetone and alcohol for 15 minutes each. After cleaning, both the steel sheets and aluminum alloys were dried. A total of 10 mg of MNs@PBEO was added to 10 g of waterborne acrylic latex and mixed thoroughly. The mixture was defoamed using a defoamer for 10 minutes at 1500 rpm. By adjusting the height of the coating device, the latex was applied to the Q235 carbon steel and 5052 aluminum alloys, resulting in a 0.2 wt% MNs@PBEO/acrylic composite coating after drying. Similarly, composite coatings with 0.5 wt%, 1 wt%, and 2 wt% MNs@PBEO were prepared by varying the amount of MNs@PBEO. The dry film thickness of all composite coatings on the Q235 carbon steel and 5052 aluminum alloy are 120±10 μm.

1.6 Characterizations

The morphology of samples was observed using scanning electron microscopy (SEM, JEOL JSM-IT200A, JPN). The chemical composition of samples was characterized by Fourier transform infrared spectroscopy (FTIR, Spectrum100, USA) and X-ray photoelectron spectroscopy (XPS, SHIMADZU AXIS SUPRA + X, JPN). A thermal gravimetric analyzer (TGA, NETZSCH TG209F1 Libra, GER) was used to evaluate the thermal properties under the N_2 atmosphere within the temperature range from 25°C to 800°C with a heating rate of 10 °C/min. The water contact angles (WCAs) of the coatings were determined using a contact angle device (OCA15EC, GER) with three measurements per sample. The adhesion of the coatings was tested by automatic pull-off adhesion tester (BEVS2201, CHINA) following ASTM D4541 at 0.5 MPa/s loading rate with three replicates. Energy-dispersive X-ray spectroscopy (EDS) was selected to analyze the elements on the surface of the steel plate after pulling off the coating. The ball mill treatment using Planetary Ball Mill (QM − 3SP, Nanjing Nanda Instrument Co., Ltd.). Ultrasonic treatment using Ultrasonic cell crusher (JY92-IIDN, Ningbo Xinzhi Biotechnology Co., Ltd.).

Open circuit potential (OCP) and electrochemical impedance spectra (EIS) were measured on an electrochemical workstation (GAMRY 1010, USA) after immersion of the coatings in 3.5 wt% NaCl solution. EIS was carried out with a standard three-electrode system: a saturated Ag/AgCl electrode as the reference electrode, a platinum plate electrode as the counter electrode, and the coated steel sheet as a working electrode with a surface area of 4.9 cm^2. Electrochemical impedance tests are recorded in the frequency range from 10^5 Hz to 10^{-2} Hz with an AC amplitude of 20 mV. Before each electrochemical measurement, the coating samples need to be placed in 3.5 wt% NaCl solution for 30 min to set up a stable state. The EIS data are analyzed by the ZsimpWin software. Among them, capacitance and diffusion coefficients were calculated from three independent EIS measurements.

All quantitative results represent mean values ± standard deviation (SD) from three independent replicates (n=3).

2. Supporting Figures

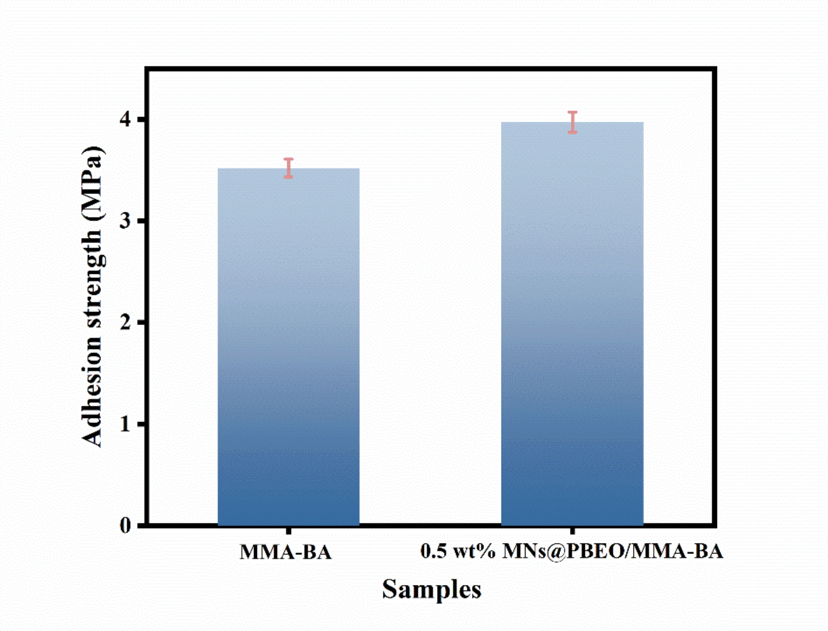

Figure S1. Adhesion tests of waterborne acrylic and MNs@PBEO/acrylic coatings coated on Q235 carbon steel [Error bars: ± SD, n=3].

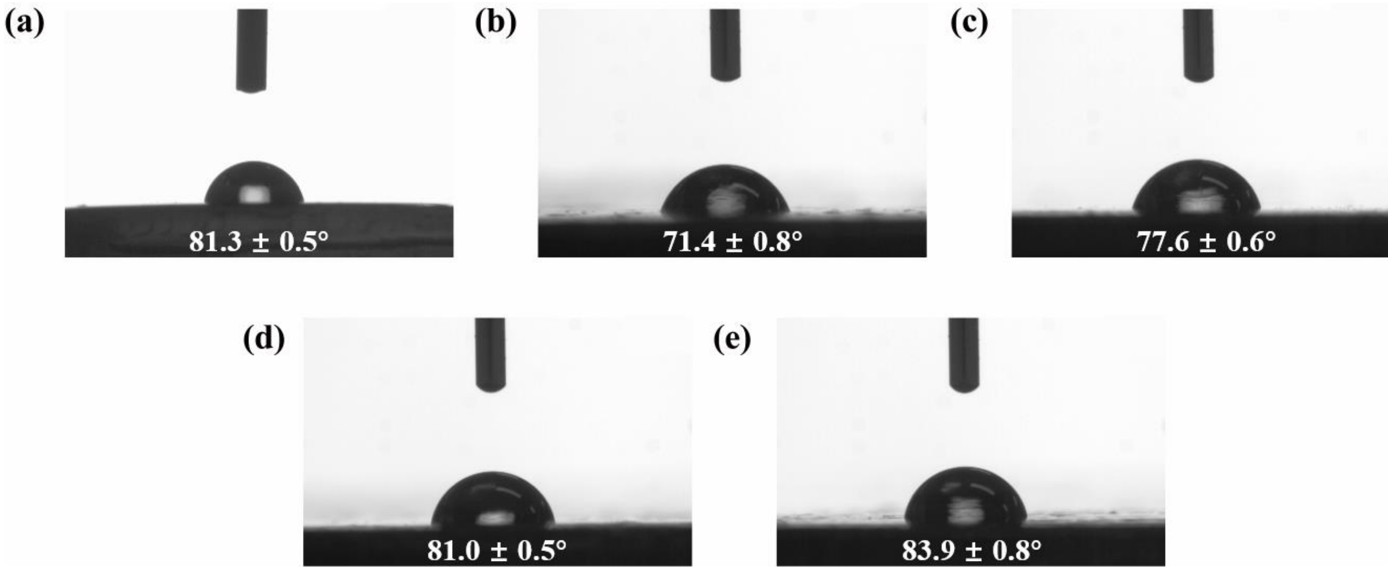

Figure S2. Water contact angles of MNs@PBEO/MMA-BA coatings with different mass fractions of (a) 0 wt%; (b) 0.2 wt%; (c) 0.5 wt%; (d) 1 wt%; (e) 2 wt% [Data format: Mean ± SD].

Table S1 Content of major elements in XPS of MNs and MNs@PBEO (with * is the sample after PBEO modification).

elements	Atomic %
O_{1s}	65.24
C_{1s}	11.08
P_{2p}	0.24
O_{1s}*	47.5
C_{1s}*	45.21
P_{2p}*	7.29
